# Coherent momentum control of forbidden excitons

**DOI:** 10.1038/s41467-022-34740-5

**Published:** 2022-11-14

**Authors:** Xuezhi Ma, Kaushik Kudtarkar, Yixin Chen, Preston Cunha, Yuan Ma, Kenji Watanabe, Takashi Taniguchi, Xiaofeng Qian, M. Cynthia Hipwell, Zi Jing Wong, Shoufeng Lan

**Affiliations:** 1 Department of Mechanical Engineering, Texas A&M University, College Station, TX 77843 USA; 2 Institute of Materials Research and Engineering, Agency for Science, Technology and Research (A*STAR), Singapore, Singapore; 3 Department of Aerospace Engineering, Texas A&M University, College Station, TX 77843 USA; 4 Department of Materials Science and Engineering, Texas A&M University, College Station, TX 77843 USA; 5 Department of Mechanical Engineering and Research Institute for Intelligent Wearable Systems, The Hong Kong Polytechnic University, Hong Kong, China; 6 Research Center for Functional Materials, National Institute for Materials Science, Tsukuba, Japan; 7 International Center for Materials Nanoarchitectonics, National Institute for Materials Science, Tsukuba, Japan; 8 Department of Physics and Astronomy, Texas A&M University, College Station, TX 77843 USA; 9 Department of Electrical and Computer Engineering, Texas A&M University, College Station, TX 77843 USA

**Keywords:** Photonic crystals, Nanophotonics and plasmonics

## Abstract

A double-edged sword in two-dimensional material science and technology is optically forbidden dark exciton. On the one hand, it is fascinating for condensed matter physics, quantum information processing, and optoelectronics due to its long lifetime. On the other hand, it is notorious for being optically inaccessible from both excitation and detection standpoints. Here, we provide an efficient and low-loss solution to the dilemma by reintroducing photonics bound states in the continuum (BICs) to manipulate dark excitons in the momentum space. In a monolayer tungsten diselenide under normal incidence, we demonstrated a giant enhancement (~1400) for dark excitons enabled by transverse magnetic BICs with intrinsic out-of-plane electric fields. By further employing widely tunable Friedrich-Wintgen BICs, we demonstrated highly directional emission from the dark excitons with a divergence angle of merely 7°. We found that the directional emission is coherent at room temperature, unambiguously shown in polarization analyses and interference measurements. Therefore, the BICs reintroduced as a momentum-space photonic environment could be an intriguing platform to reshape and redefine light-matter interactions in nearby quantum materials, such as low-dimensional materials, otherwise challenging or even impossible to achieve.

## Abstract (web summary)

Here, the authors integrate a photonic crystal, supporting photonic bound states in the continuum (BICs), with monolayer WSe_2_, and leverage the high energy confinement of the BIC modes to demonstrate coherent directional dark exciton emission.

## Introduction

Dark excitons (X_D_) in semiconductors have a long lifetime due to their decoupling from radiative channels and spin-flip processes^1–5^, making them perfect to serve as the quantum bits (qubits) for the ongoing development of quantum computing^6^. Dark excitons in two-dimensional (2D) semiconductors, such as transition metal dichalcogenide (TMD) monolayers, have also attracted broad research interests thanks to the atomically thin structure of 2D materials that made them suitable for compact planar devices^7^. However, since the excitonic transition of dark excitons does not satisfy the selection rules and possesses zero in-plane dipole moments^3,8,9^, achieving dark exciton optical brightening with conventional far-field optical techniques has been a challenging task^10^. Additionally, collecting X_D_ emission requires a large-numerical-aperture (NA) objective lens due to their out-of-plane dipole-like radiation pattern, which made it challenging to achieve information read-out using conventional far-field optical techniques. To demonstrate a robust dark exciton qubit read-out system, two tasks need to be satisfied, namely dark exciton optical brightening, and efficient collection of the X_D_ emission.

For dark exciton brightening, firstly, the current techniques to excite the X_D_ emission usually require an ultra-strong in-plane magnetic field (>14 T)^2^ or out-of-plane polarized surface plasmon polaritons (SPPs)^3^. The former brightening method uses an ultra-strong in-plane magnetic field to tilt the effective internal magnetic field in the conduction band (CB) to gain an in-plane component from the original out-of-plane electron spin. The latter method uses the SPP structure to convert the in-plane polarized incident light into an out-of-plane SPP mode to efficiently couple with the out-of-plane transition dipole moment of dark excitons. In addition, out-of-plane transition dipole moments of dark excitons in WSe_2_ monolayer encapsulated between thin h-BN flakes can couple with out-of-plane polarized incident light provided by a 90° rotated objective lens, i.e., the dark excitons can be excited by the horizontally oriented objective lens^10^. These methods, however, are intrinsically restricted to cryogenic temperature conditions (*T* < 30 K). Alternatively, a tip-enhanced photoluminescence (TEPL) system manifested itself as capable of brightening dark excitons at room temperature^4^. The oblique incident laser can be coupled in, and its out-of-plane polarized component can be selectively enhanced by the scanning probe microscope (STM) driven gap mode between the gold tip and gold substrate, yielding a greater coupling efficiency with the out-of-plane transition dipole moment of dark excitons. However, its complicated setup limits its application, especially when integrating the dark exciton read-out system with an on-chip systems^11^.

Secondly, efficient X_D_ emission collection is another bottleneck for the qubits read-out system. A nanoscale trench coupler^3^, waveguide-based method^9^, or oblique collecting setups^4^ are usually used to boost the collection efficiency of X_D_ emissions. However, the complicated setup, relatively low collection efficiency, or the absence of the enhancement of X_D_ emission limit their applications. On the other hand, optical resonators such as photonic crystal cavities^12^, whispering gallery mode (WGM) resonators^13^, antenna array Mie resonators^14^, and metamaterials^15^ are used to enhance and directionally emit the electroluminescence (EL) or photoluminescence (PL) signals from bright excitons. To combine those advantages, designing a structure that can achieve the directional X_D_ emission with suitable deflection angles can break the bottleneck for the X_D_ read-out system. In this way, X_D_ emission can be easily detected by either a conventional microscope or a single detector. Practically, recently emerged optical bound states in the continuum (BICs) with infinite quality factors (Q-factors) or quasi-BICs with finite Q-factors stand out from optical resonators for this mission, thanks to their high Q-factor and compatibility with planar optical platforms. These BICs can be supported in various photonic systems such as photonic crystal slabs^16^, plasmonic structures^17^, metasurfaces^18^, and fiber Bragg gratings^19^. BICs can be assigned to three categories: (i) symmetry-protected BICs at Gamma-points (Γ-points, the center of the Brillouin zone), (ii) off-Γ accidental BICs, and (iii) Friedrich-Wintgen BICs^20,21^. In 1985, Friedrich and Wintgen suggested that BIC can occur due to the interference of resonances belonging to different channels that cause an avoided crossing^22^. The avoided crossing was then extended to acoustic^23^, quantum^24^ and optical systems^25^. Compared with accidental BICs, which rely on carefully tuning the geometric parameters, Friedrich-Wintgen BICs are stable and efficient, making them perfect candidates for X_D_ directional emission^26,27^.

**Fig. 1:**
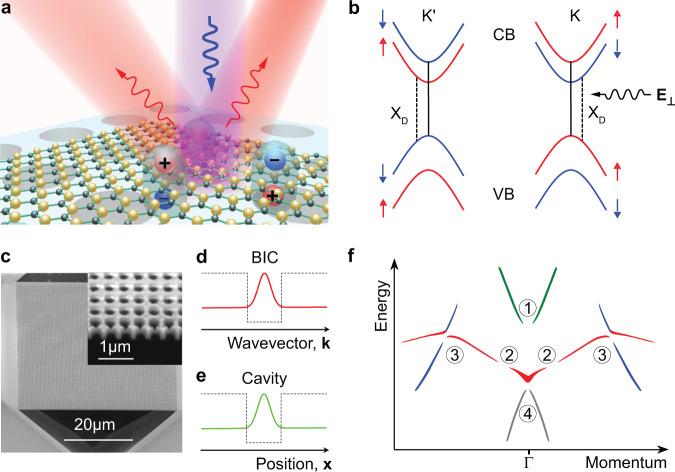
Dark exciton brightening and directional emission. **a** Schematic of directional emission of dark excitons in the WSe_2_ monolayer with normally incident pumping light. The dark excitons PL signal can then be directionally emitted through the Friedrich-Wintgen bound states in the continuum (BIC) supported by the same PhC slab. **b** Split-band configuration of bright and dark exciton states. The spin-forbidden optical transition of the dark exciton (X_D_) is brightened by the converted ${{{{{{\bf{E}}}}}}}_{{{{{{\boldsymbol{\perp }}}}}}}$ with enhancement on the top surface of the PhC slab. CB, conduction band; VB, valence band. **c** A scanning electron microscope (SEM) image of the PhC slab made of silicon nitride (Si_3_N_4_). **d** and **e** BIC, and cavity modes obtain optical confinement in momentum (**k**) and real (**x**) space, respectively. **f** A sketch of the optical band structure of the PhC slab with three types of the BICs: ① and ④ are the on-Γ symmetry-protected BICs, ② is the off-Γ accidental BIC, and ③ is the Friedrich-Wintgen BIC due to the destructive interference of resonances belonging to different bands (red and blue).

In the present work, we designed a suspended photonic crystal (PhC) slab made of lossless silicon nitride (Si_3_N_4_) which simultaneously supports on-Γ symmetry-protected BICs and off-Γ Friedrich-Wintgen BICs (Fig. 1a). In this design, a transverse magnetic (TM) like BIC at the Γ-point can efficiently convert the in-plane polarized normally-incident pump laser into out-of-plane polarized near-field energy to gain significant efficiency to couple with the out-of-plane transition dipole moment of dark excitons^11,16,28^. In this way, the spin-forbidden electrons transition from the valence band (VB) to the conduction band (CB) with opposite spin direction can be allowed, resulting in dark exciton brightening (Fig. 1b, the X_D_ transitions show this process). Meanwhile, the off-Γ Friedrich-Wintgen BIC can selectively couple with the out-of-plane dipole moment of dark excitons and directionally emit the X_D_-PL signals^29,30^. Unlike the regular bound states, which are confined in real space and rely on the well-like structure to achieve the confinement (Fig. 1d), the BICs are confined in the momentum space with infinite Q-factors and can get rid of the well-like structures, making the on-chip applications possible^20^.

## Results

In the experiments, we transferred exfoliated monolayers of WSe_2_ onto the suspended Si_3_N_4_ PhC slab device (Supplementary Fig. 2, the optical microscope picture of the device and Fig. 1c, the scanning electron microscope image of the PhC slab, and the transfer details see Method). The sketch of the band structure of the device, as shown in Fig. 1f, was extracted from the numerically simulated angle-resolved reflection spectroscopy mapping to highlight the BICs modes supported by the designed PhC slab. It clearly shows three types of BICs: ① and ④ are on-Γ symmetry-protected BICs, ② is an off-Γ accidental BIC, and ③ is an off-Γ Friedrich-Wintgen BIC due to the destructive interference of resonances belonging to different TM-like bands (red and blue). In our device, the PhC slab fulfills two important roles simultaneously: it converts polarization and enhances the incident light, and it selectively enhances and directionally emits the X_D_-PL signal for higher X_D_ collection efficiency. Only with these two roles achieved at the same time can the WSe_2_ monolayer integrate with photonic chips for robust dark exciton qubits read-out system. In light of the relatively small emission energy difference between dark and bright excitons (~40–50 meV), a large enhancement factor of X_D_ emission, typically, is necessary to increase the dark-to-bright excitons contrast, especially at room temperature^4^. However, the lifetime of dark excitons would be largely decreased due to the Purcell effect, hindering the excitons applications, especially in a quantum system. Alternatively, we used the off-Γ Friedrich-Wintgen BIC to provide a dark-exciton emission channel to separate the dark exciton signal in momentum space for an increased dark-to-bright excitons contrast. With the PhC slab, the double BICs mediated far-field-to-near-field-to-far-field mode transformation, giving rise to a ~1400-fold X_D_-PL enhancement demonstrated in the WSe_2_ monolayer with a relatively small Purcell factor of 26.7. Furthermore, the Friedrich-Wintgen BIC can be tuned to emit the X_D_ signal in various angles from 31.4° to 59.5° by tuning the device parameters, demonstrating an angle-tunable dark exciton read-out system. Finally, we also demonstrate the X_D_ directional emissions are coherent at room temperature.

### Dark exciton brightening using on-Γ symmetry protected BIC at room temperature

To demonstrate the mechanism of dark exciton brightening by on-Γ symmetry protected BIC, we first designed a suspended Si_3_N_4_ PhC slab (*n* = 2.23, near 700 nm in wavelength, thickness is 265 nm) with a square array of cylindrical holes (periodicity is 450 nm, hole radius is 140 nm). The slab material, Si_3_N_4_, is deposited using low-pressure chemical vapor deposition (LPCVD) on top of an <100> oriented silicon wafer, providing low absorption and the ability to guide the electromagnetic energy inside. The optimized fabrication protocol (see Method) was applied to yield a PhC slab with minimum surface roughness to minimize scattering and improve the quality of the interface between the WSe_2_ monolayer and the PhC slab. The device was 100-by-100 periods ($45\times 45$ μm^2^), which was sufficiently large to guarantee the C_4_ symmetry and periodic boundary condition of the lattice in the central region of the device. Supplementary Fig. 2a shows the transferred WSe_2_ monolayer with a few-layer h-BN flake covering the suspended PhC slab. It is worth mentioning that the h-BN flake can introduce a red-shift in spectra to the PhC slab^31,32^. To compensate for this redshift, we reduced the thickness of the PhC device by several nanometers in our experiment to minimize any spectral differences between the simulation and the real device. This compensation strategy is effective, thanks to the small difference in refraction index between our Si_3_N_4_ slab (*n* = 2.23, near 700 nm in wavelength, more dispersive data see Supplementary Section 15) and the exfoliated h-BN flake (*n* = ~2.2, near 700 nm in wavelength)^33^. The demonstration of this compensation strategy can be found in Supplementary Section 17.

**Fig. 2:**
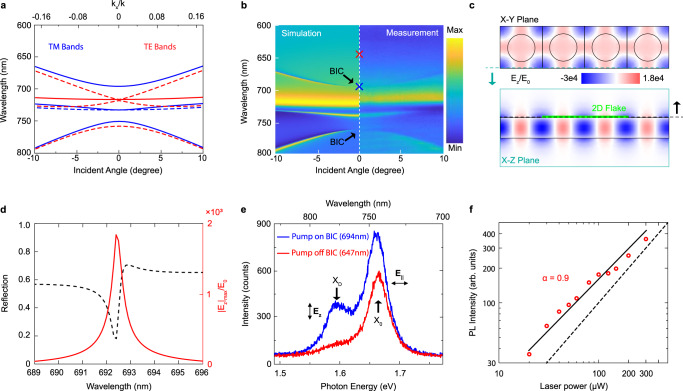
Brightening of dark excitons with BICs. **a** The band structure of a PhC slab that supports a symmetry-protected on-Γ BIC with a zero-degree incident angle. The blue bands are the transverse magnetic mode-like (TM-like) bands whereas the red bands are the transverse electric mode-like (TE-like) bands. Only four of the bands (solid lines) can be observed under p-polarized incident light. The PhC only supports the on-Γ BIC to illustrate the dark exciton brightening. **b** The simulated (left) and the measured (right) angle-resolved reflection spectra mapping of the PhC slab. It is clear to see the two on-Γ BICs at wavelengths 694 nm and 750 nm, respectively. **c** Electric-field profile ${{{{{{\bf{E}}}}}}}_{{{{{{\bf{z}}}}}}}/{{{{{{\bf{E}}}}}}}_{{{{{{\bf{0}}}}}}}$ of the on-Γ BICs, plotted on the top surface of the PhC slab (top) and the **y** = −*r*/2 slice (bottom). **d** The reflection spectrum with an oblique incident angle of 3° is shown by the black dashed line, while the maximum local electric field amplitude enhancement ratio on the top of the PhC slab is plotted in red. WSe_2_ monolayer was considered. **e** The PL spectra of dark excitons and bright excitons. The blue spectrum was taken when the pump laser matched the on-Γ BICs at the wavelength of 694 nm (on-BIC) whereas the red spectrum was taken when the pump laser was at the wavelength of 647 nm (off-BIC). **f** A log plot of the power dependence of PL intensity of dark excitons. The black line is a fit of the dark exciton emissions exhibiting a linear power dependence. The fitted slop *α* is 0.9 indicating the PL stem from dark excitons rather than bi-excitons.

To better understand the optical properties of the PhC device, we performed numerical simulations and characterizations of the device. Figure 2a shows the dispersion curves of the eight lowest energy bands (optical band structures, by the MPB band solver^34^) along the Γ-X line [${{{{{\bf{k}}}}}}(\Gamma )=(0,0)*(2\pi /a),{{{{{\bf{k}}}}}}({{{\rm X}}})=(0.5,0)*(2\pi /a),{{{{{\bf{k}}}}}}=({{{{{{\bf{k}}}}}}}_{{{{{{\bf{x}}}}}}}+{{{{{{\bf{k}}}}}}}_{{{{{{\bf{y}}}}}}})\,{{{{{\rm{and}}}}}}\,{{{{{{\bf{k}}}}}}}_{{{{{{\bf{x}}}}}}}=(\omega /c){\sin }(\theta )$]. The four blue bands are TM-like, whereas the four red bands are transverse electric (TE) like. Three TM-like bands and one TE-like band can be excited by the p-polarized incident light as shown in the mapping of the reflection spectra as a function of the incident angle in Fig. 2b. The comparison between the numerical simulation (left) and the angle-resolved spectrometric measurement (right) shows minimum differences, demonstrating the quality of the fabricated device. Two symmetry-protected TM-like BIC modes at the Γ-point at 694 nm and 755 nm are clearly visible in Fig. 2b. We designed a BIC at 694 nm as the incident light polarization converter because the BIC can convert the in-plane polarized incident light into the out-of-plane polarized component with a large enhancement ratio, as shown in Fig. 2c (the out-of-plane electric field component, ${{{{{{\bf{E}}}}}}}_{{{{{{\bf{z}}}}}}}$ distribution on the **X**-**Y** plane (upper) and the **X**-**Z** plane (lower) in the WSe_2_ monolayer). A numerical simulation using COMSOL Multiphysics shows that ${{{{{{\bf{E}}}}}}}_{{{{{{\bf{z}}}}}}}$ can be created and enhanced with an enhancement ratio reaching ~$3\times {10}^{4}$-fold at the maximum point of resonance (BIC) when compared with the original incident electrical field. In the real experiment, the tightly focused mono-color incident laser spot still has a relatively large divergent incident angle and a wide width of wavelength in the spectra. As a result, only a small part of the energy of the incident laser can couple with the BIC and be converted and enhanced, even if the 5$\times$ beam expander (Thorlabs, BE05-10-A was used to compress the beam size, more details see Supplementary Fig. 8a) and the laser line filter (Thorlabs, FL694.3-10 was placed with a small twisted angle, more details see Supplementary Figs. 8b and c) were applied to compress the laser energy into the BIC mode. Figure 2d shows the reflection spectrum with an oblique incident angle of 3° (indicated by the black dashed line) and the maximum local ${{{{{{\bf{E}}}}}}}_{{{{{{\bf{z}}}}}}}$ enhancement ratio over the incident light electric field on the top surface of the PhC slab, where the WSe_2_ monolayer is seated. However, it is clearly seen that only the laser energy that is close to the resonance peak benefits from the large enhancement factor. To quantitatively estimate the ${{{{{{\bf{E}}}}}}}_{{{{{{\bf{z}}}}}}}$ conversion and enhancement efficiency, we calculated the average ${\left({{{{{{\bf{E}}}}}}}_{{{{{{\bf{z}}}}}}}/{{{{{{\bf{E}}}}}}}_{{{{{{\bf{0}}}}}}}\right)}^{2}$as the enhancement factor (more details see Supplementary Section 8) and found that the average ${\left({{{{{{\bf{E}}}}}}}_{{{{{{\bf{z}}}}}}}/{{{{{{\bf{E}}}}}}}_{{{{{{\bf{0}}}}}}}\right)}^{2}$ is 216 when the full-width-half-maximum (FWHM) of the wavelength is 3 nm and the FWHM of the incident laser divergence angle is 4° ($\pm$ 2°), which provides sufficiently large enhancement of the out-of-plane component of the near field energy for dark exciton brightening.

The PL spectra of the WSe_2_ monolayer device were excited by laser with a wavelength of 694 nm (blue, on-BIC) and 647 nm (red, off-BIC) respectively, which are shown in Fig. 2e. We used a 40$\times$ large numerical aperture objective lens (40$\times$ Nikon S Flour high NA objective, NA = 0.9) to excite the incident laser and collect the PL signal. A strong X_D_ emission peak was observed (blue spectrum) when the incident laser matched the BIC mode, whereas the X_D_ emission channel was closed when the incident laser was off-resonance (off-BIC, red spectrum). From the PL spectra for X_D_ and bright excitons (X_0_), we obtained ~52 meV of intravalley energy splitting between the X_D_ and X_0_. This result is in good agreement with other X_D_ emission observations, namely 47 meV obtained by in-plane magnetic field^2^, 42 meV by SPP coupling^3^, and 46 meV by TEPL setup^4^. To eliminate the possibility that emission stemmed from bi-excitons, which have similar emission energy compared to dark excitons in WSe_2_^35^, we measured the PL signal intensity as a function of excitation power. The intensity of the PL emission and the excitation power are related, i.e., ${I}_{{PL}}\propto {I}_{{excited}}^{\alpha }$, where *α* is the order of the excitation intensity. From this equation, the PL emission can be recognized as bi-excitons when *α* = 2 and as bright or dark excitons when *α* = 1, respectively^35^. In a realistic experiment, the factor α would be expected in the range of 1.2–1.9 for bi-excitons, which have been extensively studied^35,36^. In our experiment, the logarithmic plot of the dark excitons PL peak with respect to the excitation power, as shown in Fig. 2f, shows the fitted exponent of *α* = 0.9, indicating the emission stemmed from dark excitons.

### Directional and coherent emission of dark excitons by Friedrich-Wintgen BIC

Because of their out-of-plane dipole radiation nature, X_D_ emits primarily towards the in-plane direction, making the PL signal difficult to be collected with conventional microscopic objective lenses^10^. Supplementary Fig. 10 shows the collection efficiency of PL emission radiated by dark excitons and bright excitons, respectively. We also provide the collection efficiency comparison of this work and other methods (Supplementary Fig. 19). It is clear to see barely any of the X_D_ emission can be collected by an objective lens with a sufficiently large numerical aperture (NA = 0.9). To gain higher dark exciton read-out efficiency, we demonstrated a directional and polarized emission channel with tunable angles for dark excitons using the Friedrich-Wintgen BICs. This directional emission channel can selectively couple with the out-of-plane dipole moment of dark excitons and enhance their directional emission. The enhanced directional emission has a small full width at half-maximum (FWHM) of ~7° divergence angle in air and a ~1400-fold total enhancement factor with linear polarization. By tuning the geometric parameters of the PhC slab, the Friedrich-Wintgen BIC can be tuned to provide different deflection angles for dark exciton emission, making dark exciton read-out possible by a single detector placed in the proper position.

**Fig. 3:**
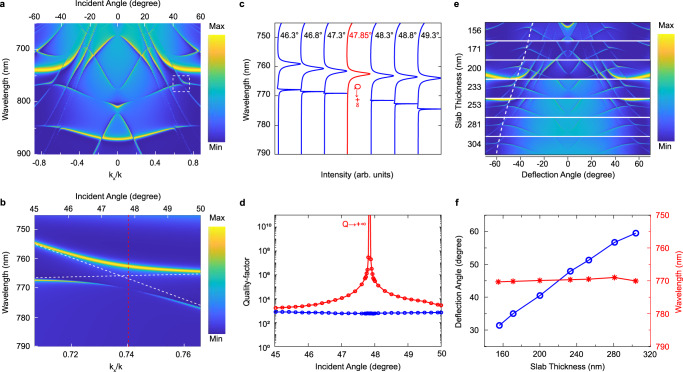
Tunable Friedrich-Wintgen BICs. **a** The dispersion spectra of the PhC that supports BICs. The Friedrich-Wintgen BIC due to the interference of two TM-like bands is highlighted by the white dashed box. **b** A close look of the Friedrich-Wintgen BIC at a wavelength of 770 nm and oblique incident angle of 47.85° (red line). The white dashed line indicates the origin of the two modes. **c** Spectra at a series of different incident angles. Avoided crossing and linewidth vanishing of the lower branch band at 47.85° are observed due to the interference between the two modes. **d** Quality factors (Q-factors) of two bands that form the Friedrich-Wintgen BIC as a function of the oblique incident angle. The blue circles and the red circles represent the Q-factors of the upper branch and the lower branch of the avoided crossing bands, respectively. Q-factors of the lower branch are rapidly increasing when the oblique incident angle approaches 47.85° where the Friedrich-Wintgen BIC occurred. **e** Friedrich-Wintgen BIC modes for X_D_ directional emission are tunable for different deflection angles. The thicker the PhC slab, the higher the deflection angle of the Friedrich-Wintgen BIC emission channel. **f** Quasi-linear relations (blue) between the deflection angle by Friedrich-Wintgen BICs and the PhC slab thickness. The Friedrich-Wintgen BIC can be tuned in the momentum space from 31.4° to 59.5° and maintain the wavelength close to ~770 nm (red).

In this experiment, the Friedrich-Wintgen BIC serves as a stable resonator with a high Q-factor and provides an efficient radiation channel for X_D_ directional emission^30,37^. We designed a suspended Si_3_N_4_ PhC slab (thickness is 233 nm) with a square array of cylindrical holes (periodicity is 510 nm, hole radius is 102 nm) that supports a TM-like symmetry-protected BIC at the Γ-point of 596 nm for dark exciton brightening (Supplementary Fig. 5) and another TM-like Friedrich-Wintgen BIC at ${{{{{{\bf{k}}}}}}}_{{{{{{\bf{x}}}}}}}$ = 0.74 (47.85°) and 770 nm for the X_D_ emission (Fig. 3a and b). Figure 3a and Supplementary Fig. 4 show the simulated and measured mapping of the reflection spectra as a function of incident angle (optical band structure), respectively. It is clear to observe the avoided crossing (with white dashed line labels) in Fig. 3a. Figure 3b shows the zoomed-in optical band structure in the Friedrich-Wintgen BIC region, indicating that the Friedrich-Wintgen BIC occurs due to the destructive interference between two TM-like bands (Mode analyses see Supplementary Fig. 7). In the reflection spectra (Fig. 3c), each mode can be described by an asymmetrical Fano line shape. Vanishing of the Fano lineshape occurred at the lower branch at 47.85° indicating that the BIC emerged with an infinite Q-factor via the destructive interference of the two resonances. We used a numerical simulation (COMSOL Multiphysics), eigenfrequency model, to extract the Q-factors along each of the two bands as shown in Fig. 3d. The Q-factor of the upper branch remains relatively low (~10^3^) whereas the Q-factor of the lower branch has an infinite peak at around 47.85°.

In addition, the Friedrich-Wintgen BICs are momentum tunable, and thus we can control the X_D_ emission towards different deflection angles. We designed a series of the Friedrich-Wintgen BICs at the wavelength of 770 nm in Fig. 3e. This momentum tunability of the dark exciton’s directional emission channel, i.e., the Friedrich-Wintgen BIC, can also benefit dark exciton information read-out using a single detector. The Friedrich-Wintgen BICs stem from the full destructive interference of two TM bands and thus are stable and easier to design in comparison with the off-Γ accidental BIC. We found that the avoided crossing of the two TM bands, exactly where the Friedrich-Wintgen BIC occurs, can be tuned by varying the PhC slab’s thickness. It is clear to see, as shown in Fig. 3e, the avoided crossing moved towards a higher angle in the momentum space as the slab thickness increased. To match the dark exciton energy (770 nm in wavelength), we changed the periodicity and as well as the radius of holes as compensation for each PhC design. The continuum tunable angle ranges from 31.4° to 59.5° as we demonstrated for some discrete cases. The white dashed line traces the avoided crossing to show the tuning trend of the Friedrich-Wintgen BICs. We further plotted the deflection angle by Friedrich-Wintgen BICs as the function of the PhC slab thickness. The relationship was quasi-linear, implying that we can extend the tunable deflection angle to a broader range.

**Fig. 4:**
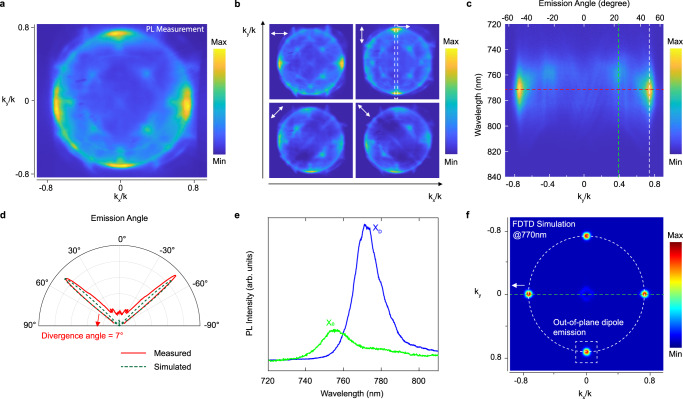
Directionality control of dark excitons. **a** PL emission momentum distribution mapping with ${{{{{{\bf{k}}}}}}}_{{{{{{\bf{x}}}}}}}$ and ${{{{{{\bf{k}}}}}}}_{{{{{{\bf{y}}}}}}}$ axis. Four shining emission spots, located at the Γ-X line and ${{{{{{\bf{k}}}}}}}_{{{{{{\bf{x}}}}}}}$ or ${{{{{{\bf{k}}}}}}}_{{{{{{\bf{y}}}}}}}$= 0.74 with C_4_ symmetry, are clearly visible. **b** Polarization analysis of the PL emission momentum distribution in **a**. The four shining emission spots show radial polarization indicating they are from dark excitons. **c** Angle-resolved PL emission spectra mapping extracted from y-polarized PL emission momentum distribution mapping in **b**. It is clear to see the dark exciton directional emissions have small divergence angles at wavelengths of around 772 nm and towards oblique emission angles of around 48°. **d** The measured (red solid line) and FDTD simulated (dark green dashed line) PL intensity as a function of the in-plane momentum (${{{{{{\bf{k}}}}}}}_{{{{{{\bf{y}}}}}}}/{{{{{\bf{k}}}}}}$) along the y-direction. The full-width-half-maximum (FWHM) of the measured X_D_ emission lobes is 7° indicating the ultra-low divergence angle of the directional emission. **e** Spectra extracted from oblique angles of 48° and 23° for the dark exciton emission and the bright exciton emission, respectively. **f** The simulated PL emission momentum distribution by the Lumerical FDTD. Four shining emission spots show high correspondence to the measured PL emission pattern in **a**.

The directional and polarized emission of dark excitons through the Friedrich-Wintgen BIC has been analyzed in Fig. 4. The few-layer h-BN flake and monolayer WSe_2_ heterostructure were sequentially transferred onto the PhC slab that supported the Friedrich-Wintgen BIC and pumped by a wavelength-tunable femtosecond-ultrafast-laser with the power of 50 μW with p-polarization. Figure 4a shows the PL emission momentum distribution mapped with ${{{{{{\bf{k}}}}}}}_{{{{{{\bf{x}}}}}}}$ and ${{{{{{\bf{k}}}}}}}_{{{{{{\bf{y}}}}}}}$ axis. Four shining emission spots, located at the Γ-X line with ${{{{{{\bf{k}}}}}}}_{{{{{{\bf{x}}}}}}}/{{{{{\bf{k}}}}}}$ or ${{{{{{\bf{k}}}}}}}_{{{{{{\bf{y}}}}}}}/{{{{{\bf{k}}}}}}$ = 0.74 and with C_4_ symmetry, are clearly visible. The PL emission momentum distribution was collected by a sensitive silicon camera (Princeton Instruments PIXIS 400) that was placed in the **k**-plane (see Method). It is worth noting that both X_0_ and X_D_ emissions were collected by the camera at the same time. To elucidate the PL emission momentum distribution in further depth, the polarization-resolved images are presented in Fig. 4b. The four shining spots were radially polarized and exhibited similar intensities. Three pieces of evidence are present to explain why the origin of these four shining spots comes from the dark excitons. 1) Supplementary Fig. 6 shows the PL emission polarization analysis of the bright or dark excitons, and it shows that only dark excitons’ out-of-plane dipole radiation nature can provide the emission with C_4_ symmetry (radial polarization); 2) The four shinning spots have similar emission intensity. If those spots came from bright excitons with p-polarized (along with the **y**-direction) in-plane momentum, the spots along the **x**-direction should be brighter than those along the **y**-direction. This argument has already considered the depolarization effect of bright exciton at room temperature (more details see Supplementary Section 12); 3) Only dark excitons with out-of-plane dipole momentum have high coupling efficiency with the Friedrich-Wintgen BIC that comes from the interference between two TM-like bands. (For the **E**-field analysis of the BIC mode see Supplementary Fig. 7). Furthermore, the polarization for each directional emission spot is linear and the extinction ratio is relatively high (shining spot can be fully blocked under cross-polarizer), proving its coherence at room temperature^38^.

The angle-resolved PL emission spectra mapping extracted from the PL emission momentum distribution with p-polarization (**y**-direction) by the diffraction grating of the spectrometer is shown in Fig. 4c. The enhanced X_D_ emission located at ${{{{{{\bf{k}}}}}}}_{{{{{{\bf{y}}}}}}}/{{{{{\bf{k}}}}}}$ = $\pm$ 0.74 at 772 nm with a small divergence angle is clearly visible (~7°, Fig. 4d). We further extracted the spectra from the angle-solved PL emission as shown in Fig. 4e, the blue line of which shows the X_D_ emission spectrum extracted from ${{{{{{\bf{k}}}}}}}_{{{{{{\bf{y}}}}}}}/{{{{{\bf{k}}}}}}$ = 0.74 and the red line shows the bright excitons emission spectrum from ${{{{{{\bf{k}}}}}}}_{{{{{{\bf{y}}}}}}}/{{{{{\bf{k}}}}}}$ = 0.39. This is clear evidence that the PhC slab can separate the emission from bright and dark excitons and selectively enhance the X_D_ emission at a specified angle with a small divergence angle.

To further understand the coupling mechanism between the out-of-plane dipole and the Friedrich-Wintgen BIC supported by the PhC slab, a Finite-difference time-domain (FDTD) simulation was performed using commercial software (Lumerical FDTD Solutions, ANSYS Inc.) to show how the PhC slab selectively couples with the out-of-plane dipole and enhances their emission. A series of out-of-plane dipoles with excited wavelengths near 770 nm (on-resonance of the Friedrich-Wintgen BIC) were randomly placed on the top surface of the PhC slab (30-by-30 periods with scattering boundary conditions). Figure 4f shows the simulated out-of-plane dipole emission momentum distribution, which perfectly matched the X_D_ emission part of the PL emission momentum distribution (Fig. 4a). The broadening of the emission features in the azimuthal direction of the X_D_ emission can be attributed to the thermal expansion at room temperature.

We also quantitatively estimate the enhancement factor (*EF*) of the X_D_ emission by the Friedrich-Wintgen BIC performed by the FDTD simulation (more details see Method). The enhancement factor equation is ${EF}={\left|\frac{{{{{{{\bf{E}}}}}}}_{{{{{{\bf{z}}}}}}}}{{{{{{{\bf{E}}}}}}}_{{{{{{\bf{0}}}}}}}}\right|}^{2}\times {\gamma }_{{PF}}\times \eta$, where ${\gamma }_{{PF}}$ is the Purcell factor of the Friedrich-Wintgen BIC, and the $\eta$ is the modal overlap (also known as the mode-matching factor) between the on-Γ BIC and FW-BIC. We used the FDTD method to calculate the Purcell factor the highest of which is 26.7 at a wavelength of 769 nm (more details see Supplementary Section 9). Because both the monolayer WSe_2_ flake size and the tightly focused incident light spot are larger than one period of the PhC lattice (510 nm), we used the average ${\left|{{{{{{\bf{E}}}}}}}_{{{{{{\bf{z}}}}}}}/{{{{{{\bf{E}}}}}}}_{{{{{{\bf{0}}}}}}}\right|}^{2}$ enhancement ratio instead of the near-field **E**-field enhancement ratio at any local point to estimate the *EF*. The average ${\left|{{{{{{\bf{E}}}}}}}_{{{{{{\bf{z}}}}}}}/{{{{{{\bf{E}}}}}}}_{{{{{{\bf{0}}}}}}}\right|}^{2}$ enhancement ratio by the on-Γ BIC at 596 nm is 116 (more calculation details see Supplementary Section 8) and the modal overlap is 45.73% (more calculation details see Supplementary Section 18). The *EF* is estimated to be as high as ~1400 at a wavelength of 769 nm.

### Coherence demonstration of directional emission from dark excitons

**Fig. 5:**
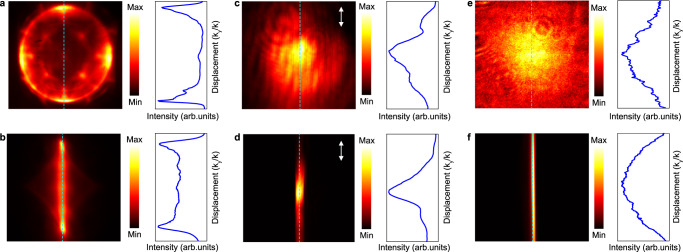
Room-temperature coherence of the directional emission. Momentum distribution and spatial interference enabled by a cylindrical lens for the directional emission of dark excitons supports an interference pattern **b**. The destructive interference in the middle of **b** is due to a *π*-phase shift between the left and right parts of the light field in **a**, showing that the directional emission is strongly coherent at room temperature. Momentum distribution and spatial interference for a coherent laser beam. The center of **d** is brighter than that of **c**, which is caused by constructive interference. Momentum distribution and spatial interference for incoherent bright exciton emission of monolayer WSe_2_ on SiO_2_/Si substrate. The intensity profile of **f** is similar to that of **e** because the bright exciton lots its valley coherence at room temperature.

Due to the long lifetime of the dark exciton and the high Purcell factor of the Friedrich-Wintgen BIC cavity, the directional emission of the dark excitons in monolayer WSe_2_ maintains their coherent nature^39^. To demonstrate this, we used a cylindrical lens to focus the emission pattern to observe its interference pattern^40,41^(more details see Supplementary Section 12). Before the coherence demonstration, we used a laser spot of Gaussian shape, reflected by a silicon wafer to observe the bright exciton emission pattern in **k**-space from monolayer WSe_2_ on a SiO_2_/Si substrate to demonstrate this method’s reliability. Figure 5a, c, and e respectively show the **k**-space images (left) and the intensity profiles (right) of the X_D_ directional emission, laser spot, and bright exciton emission, whereas Fig. 5b, d, and f show their corresponding cylindrically focused patterns (left) and intensity profiles (right). Because the bright exciton emission would lose its valley coherence at room temperature and shows nearly random polarization, the intensity profile (labeled by a dashed line) of the **k**-space pattern (Fig. 5e, right) and the cylindrically focused pattern (Fig. 5f, right) should be Gaussian-like^4^. On the other hand, the laser spot has naturally good coherence and as a result, its intensity profile of the cylindrically focused pattern (Fig. 5c, right) should have a narrower FWHM because of the constructive interference of the common phase. Finally, we cylindrically focused the dark exciton directional emission pattern into one dimension, meaning the left and right emission spots overlap. Because they (the left and right spots) have the same polarization and a phase difference of *π* (C_4_ symmetry of this system) as we discussed in the manuscript, the destructive interference pattern in the middle is expected (Fig. 5b).

## Discussion

We designed a suspended Si_3_N_4_ PhC slab that simultaneously supports a TM-like on-Γ symmetry-protected BIC and a tunable off-Γ Friedrich-Wintgen BIC. The supported on-Γ BIC can efficiently convert the in-plane polarized normal incident pump laser into out-of-plane polarized near-field energy to gain large efficiency as high as 116 to couple with the out-of-plane transition dipole moment, thus brightening the spin-forbidden dark excitons in monolayer WSe_2_ at room temperature. We also demonstrated that the pump laser could regulate the X_D_ emission by switching frequencies on- or off- the BIC mode. Further, the off-Γ Friedrich-Wintgen BIC can selectively couple with the out-of-plane transition dipole moment of dark excitons and provide an efficient directional and polarized emission channel for the dark excitons with minimized divergence angle (FWHM of ~7° in the air) and ~1400-fold enhancement factor. By tuning the thickness of the PhC slab, the Friedrich-Wintgen BIC can be tuned to emit the dark excitons signal at various angles from 31.4° to 59.5°. Furthermore, we demonstrated the coherence of the X_D_ directional emission at room temperature by the constructive interference of the directional emissions that towards opposite directions. The dark exciton brightening and directional and coherent emission by a planar device demonstrate a robust dark exciton information read-out system, paving the way for on-chip computing and communications.

## Methods

### Sample fabrication

A 300 nm thick layer of Si_3_N_4_ was deposited on Si substrates using low-pressure chemical vapor deposition (LPCVD). The periodic PhC pattern with a periodicity of 465 nm (or 510 nm) and hole radius of 140 nm (or 102 nm) was written on ZEP-520A photoresist using the TESCAN MIRA3 E-beam lithography system. Then the RIE-ICP cyclic dry etch strategy was applied to transfer the pattern from the photoresist to the Si_3_N_4_ layer. After removing the ZEP-520 photoresist residual with hot acetone (90 °C) and an oxygen plasma cleaning procedure, the silicon (<100> oriented) beneath the PhC pattern was undercut using KOH solution (30 wt%) at 120 °C to suspend the PhC slab. Finally, modified RIE etching was applied without a mask to reduce the thickness of the slab to 265 nm (or 233 nm).

Few-layers h-BN flakes and monolayer WSe_2_ samples were mechanically exfoliated from bulk crystals onto 295 nm thick SiO_2_/Si wafers. WSe_2_ monolayers were identified under an optical microscope and verified using photoluminescence measurements. Few-layer h-BN flakes and WSe_2_ monolayer samples were transferred onto suspended photonic crystal devices using the capillary-force-assisted clean-stamp transfer method^42^.

### Optical measurement

The Fourier-optics-based spectroscopy has three operating modes: an imaging mode, an optical band-structure (angle-resolved reflection spectroscopy) mode, and a spectra analysis mode. For the imaging mode, a broadband emission halogen lamp (QTH10, Thorlabs) with a wavelength range of 400–2200 nm was used to provide white light. A CCD camera (16MP high-speed USB 3.0 digital camera, AmScope) was placed at the image plane after the tube lens to show the image (Supplementary Fig. 3). For the optical band-structure (angle-resolved reflection spectroscopy) mode, the back focal plane of an objective lens (20$\times$ Mitutoyo Plan Apo Infinity Corrected Long WD objective, NA = 0.42 or 40$\times$ Nikon S Flour high NA objective, NA = 0.9) was projected by a Fourier transform system. A pinhole was placed at the image plane after the tube lens (*f* = 200 mm) to regulate the field of view for the light reflected from the photonic crystal slab. A slit spectrometer (Princeton Instruments, spectrometer SP300, and silicon camera PIXIS 400) was placed at the K-plane and switched to spectrometer mode for the optical band-structure or imaging mode for **k**-space imaging. For the spectra analysis mode, the Fourier lens L4 (*f* = 100 mm) was replaced with an imaging lens L3 (*f* = 50 mm) to Fourier transform the **k**-plane into an image plane. The spectrometer can be used to collect the spectra in the image plane (Supplementary Fig. 3b).

Photoluminescence spectroscopy can be achieved by switching flip mirror to the laser mode. The sample was pumped by a wavelength-tunable mode-locked Ti: Sapphire laser (Chameleon Ultra II, Coherent) and a cascade of OPO (optical parametric oscillator). A high numerical aperture (NA = 0.9) 40$\times$ objective lens (40$\times$ Nikon S Flour high NA objective) was used to collect the PL signal at room temperature. The PL signal was then sent to the **k**-plane by the Fourier transform system and imaged at the spectrometer for either **k**-plane imaging or the PL emission momentum distribution. A short pass filter (FESH0700, Thorlabs) was used to clean the undesired wavelength before the pump laser reached the objective length and a long pass filter (FELH0700, Thorlabs) was used to block the pump power to make the PL signal stand out.

### Numerical simulation

Numerical simulations of the far field emission patterns and Purcell enhancement factors are carried out using three-dimensional full-wave finite-difference time-domain methods (Lumerical FDTD Solutions, ANSYS Inc.). The simulation spans in **x-** and **y-** directions encompassing a sample area of 30-by-30 periods. Perfectly matched layers (PMLs) are used as boundary conditions in all directions. The X_D_ emission is simulated by placing a **z**-oriented dipole array on the upper surface of the photonic crystal slab. The radiation phase of each dipole is set randomly. The minimum mesh size is set as 2 nm around the emitting region. To collect the emission profile, a far field pattern monitor is placed 1 μm above the sample surface. To calculate the Purcell enhancement, a control simulation with the same Si_3_N_4_ slab without the holes is carried out. The Purcell enhancement factor is calculated by extracting the emission intensity at 48° for both cases with and without lattices.

Numerical simulations of the angle-resolved reflection spectra mapping are carried out using the finite element method (COMSOL Multiphysics). We selected one unit cell as the simulation domain and use the periodic (Bloch) boundary condition in x and y directions and the periodic type of port to excite the horizontal magnetic field with various oblique incident angles for the input light. The S-parameter, S11, was used for the reflection spectra.

## Supplementary information

Supplementary Information

## Data Availability

All data that support the findings of this study are available in the main text, figures, and Supplementary Information. They are also available from the corresponding author upon reasonable request.
