# Supplementary material for: Coherent momentum control of forbidden excitons
Source: Nat Commun. 2022 Nov 14;13:6916. doi: 10.1038/s41467-022-34740-5 (PMC9663599; doi:10.1038/s41467-022-34740-5)
Supplement: Supplementary file 1 — Supplementary Information [file 41467_2022_34740_MOESM1_ESM.pdf]

Supplementary Information for: "*Coherent momentum control of forbidden excitons*"

Xuezhi Ma[1,2], Kaushik Kudtarkar[1], Yixin Chen[3,4], Preston Cunha[1], Yuan Ma[1,5], Kenji Watanabe[6], Takashi Taniguchi[7], Xiaofeng Qian[4,8,9], M. Cynthia Hipwell[1], Zi Jing Wong[3,4], and Shoufeng Lan[1,4*]

1 Department of Mechanical Engineering, Texas A&M University, College Station, TX 77840, USA.

2 Institute of Materials Research and Engineering, Agency for Science, Technology and Research, Singapore

3 Department of Aerospace Engineering, Texas A&M University, College Station, TX 77840, USA.

4 Department of Materials Science and Engineering, Texas A&M University, College Station, TX 77840, USA.

5 Department of Mechanical Engineering, The Hong Kong Polytechnic University, Hong Kong, China.

6 Research Center for Functional Materials, National Institute for Materials Science, Tsukuba, Japan.

7 International Center for Materials Nanoarchitectonics, National Institute for Materials Science, Tsukuba, Japan.

8 Department of Physics and Astronomy, Texas A&M University, College Station, TX 77840, USA.

9 Department of Electrical and Computer Engineering, Texas A&M University, College Station, TX 77840, USA.

Author e-mail address: shoufeng@tamu.edu

Table of Contents

1. Q-factor of the on-Γ BIC for out-of-plane electric field conversion. 3
2. Optical microscope images of the devices 3
3. Optical measurement setups 4
4. The optical band structure of the photonic crystal slab given by numerical simulation and optical measurement 4
5. The on-Γ BIC for dark exciton brightening in the photonic crystal device that supported the avoid-crossing mode BICs. 5
6. The PL emission polarization analysis of the bright and dark excitons. 6
7. Formation of the Friedrich-Wintgen BIC 7
8. The calculation of the out-of-plane electric field conversion efficiency of the on-Γ BIC..... 8
9. The calculation of the Purcell factor of the avoid-crossing BIC 10
10. Collection efficiency of the PL emission radiated by bright excitons and dark excitons.... 11
11. Bright exciton PL emission decoherence at room temperature 11
12. Interference setup using a cylindrical lens 12
13. Optical band structure measurements using halogen lamp and supercontinuum laser........ 13
14. Microscopy image of the tightly focused gaussian spot 14
15. The measured refractive index of LPCVP Si_3N_4 film. 15
16. Influence of the WSe_2 monolayer 15
17. Influence of h-BN flake and compensation strategy... 18
18. Modal overlap between on-Γ BIC and FW-BIC.. 19
19. Comparison of different dark excitons brightening methods. 19

1. Q-factor of the on-Γ BIC for out-of-plane electric field conversion.

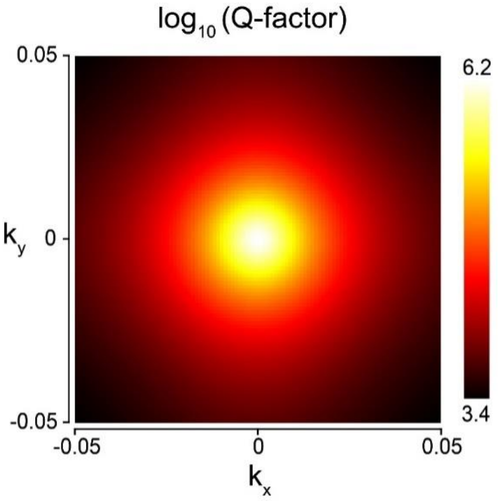

Supplementary Fig. 1: Map of the Q-factor of the on-Γ BIC at 694 nm of the photonic crystal slab device (periodicity, a = 450 nm, the radius of holes, r = 140 nm, thickness of the slab, h = 265 nm, the refractive index of the slab materials, Si_3N_4, n = 2.23).

2. Optical microscope images of the devices

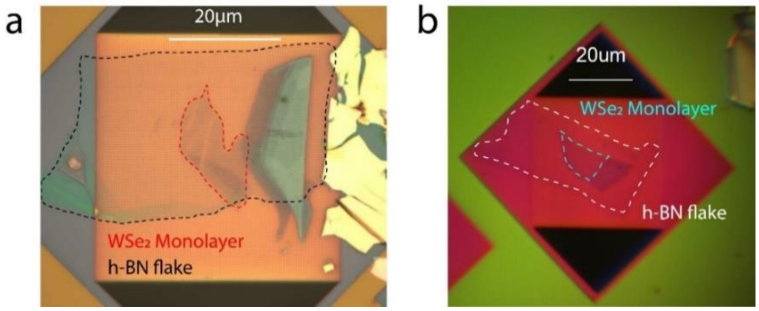

Supplementary Fig. 2: Optical microscope images of the devices. The few-layer h-BN flake and WSe_2 monolayer were transferred on the suspended photonic crystal slabs with parameters of (a) periodicity, a = 450 nm, radius of holes, r = 140 nm, and thickness of the slab, h = 265 nm, and (b) periodicity, a = 510 nm, radius of holes, r = 102 nm, and thickness of the slab, h = 233 nm, respectively.

3. Optical measurement setups

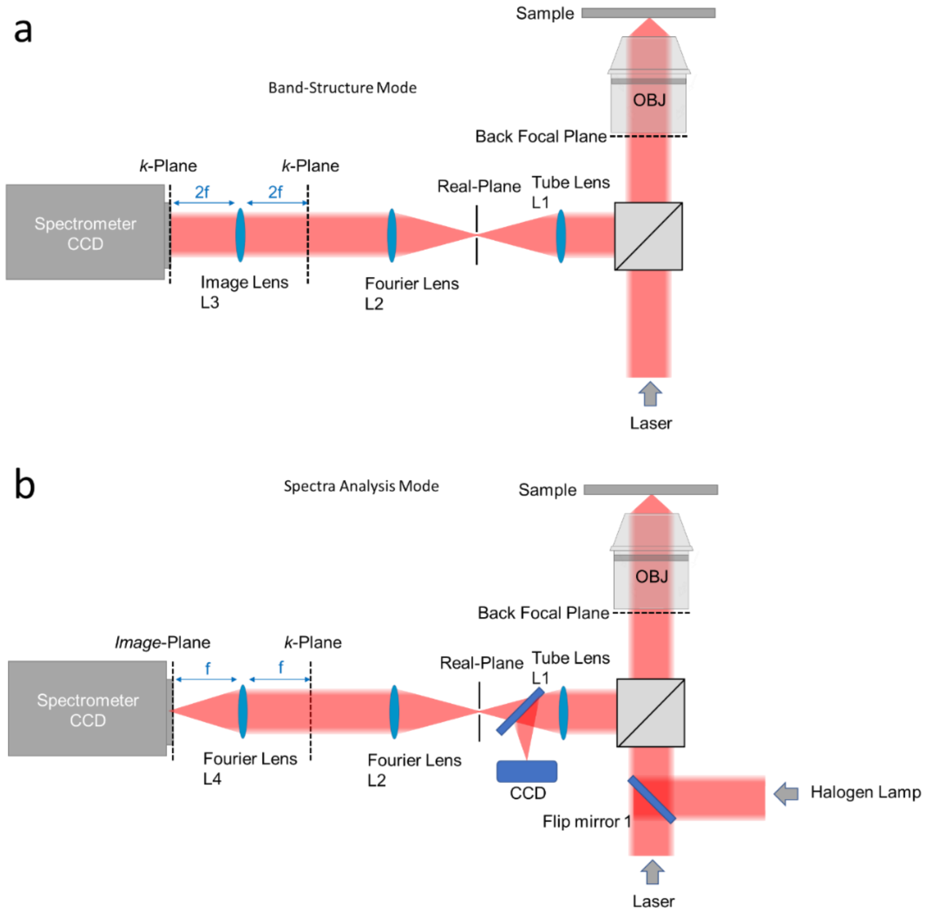

Supplementary Fig. 3: Schematic of experimental setups for optical measurements. (a) the optical band-structure mode can be achieved using an image lens L3 (f = 50 mm) to project the **k**-space image to the spectrometer. (b) the spectra analysis mode can be achieved using a Fourier lens L4 (f = 100 mm) to Fourier transfer the **k**-space image to the real-space image to the spectrometer.

4. The optical band structure of the photonic crystal slab given by numerical simulation and optical measurement

Supplementary Fig. 4 shows the optical band structures of the device with parameters of periodicity, a = 510 nm, radius of holes, r = 102 nm, the thickness of the slab, h = 233 nm. **Supplementary Fig. 4a** shows the simulated result using COMSOL whereas **Supplementary Fig.**

4b shows the measured result using our angle-resolved reflection spectroscopy. It is clear to see the two bands avoid crossing at a wavelength of 772 nm and $\mathbf{k_x/k}$ = 0.74 (the equivalent incident angle was 47.85°) at both simulated and measured optical band-structure mappings.

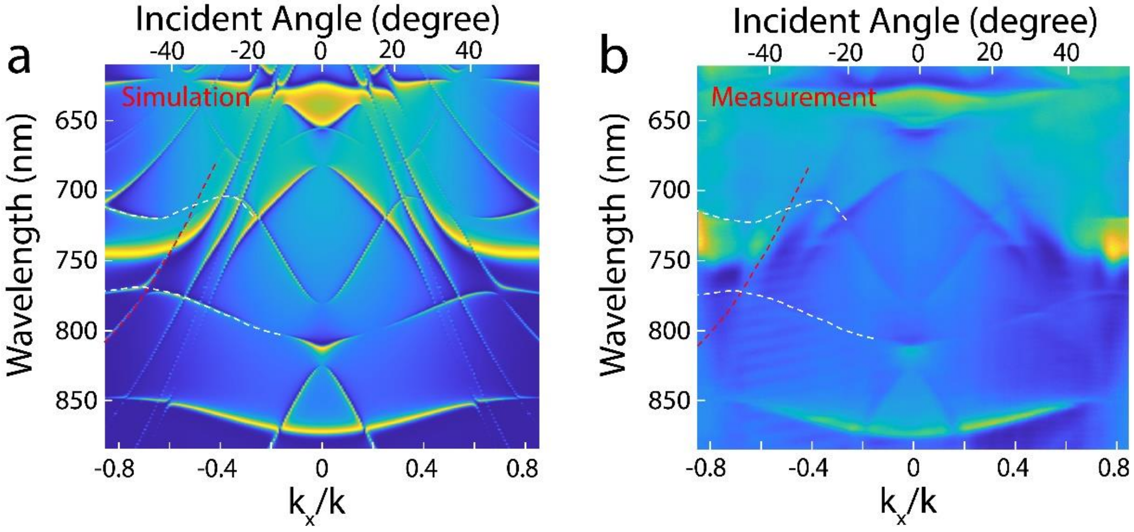

Supplementary Fig. 4: The comparison between (a) the simulated and (b) the measured optical band-structure of the photonic crystal slab with the parameters of periodicity, a = 510 nm, radius of holes, r = 102 nm, and thickness of the slab, h = 233 nm. The index of the materials (Si_3N_4) is 2.23.

5. The on-Γ BIC for dark exciton brightening in the photonic crystal device that supported the avoid-crossing mode BICs.

In the manuscript, we used two different devices, the first one supports a TM mode on-Γ BIC at a wavelength of 694 nm for dark exciton brightening and the second one supports a TM mode on-Γ BIC at a wavelength of 600 nm for dark exciton brightening and the avoided crossing mode BICs to couple the out-of-plane oriented dipole of the dark exciton and directionally emit the dark exciton PL signal. We firstly demonstrated that the TM mode on-Γ BIC can brighten the dark exciton with a device that just supports the on-Γ BIC and is left blank at the dark exciton energy. Next, the second device supported two types of different BICs and can brighten and directionally emit simultaneously.

Supplementary Fig. 5a shows the comparison of the optical band structure between the

simulation and the measurement, and they show minimal differences. It is clear to see the on-Γ BIC at a wavelength of 600 nm has a vanished line shape, indicating its infinite Q-factor. The out-of-plane oriented electric field distribution mappings in the **X-Y** plane where the transferred WSe_2 monolayer was located and, in the **X-Z** plane are shown in **Supplementary Fig. 5b.** The highly enhanced $\mathbf{E_z}$ has large efficiency in brightening the dark excitons at room temperature.

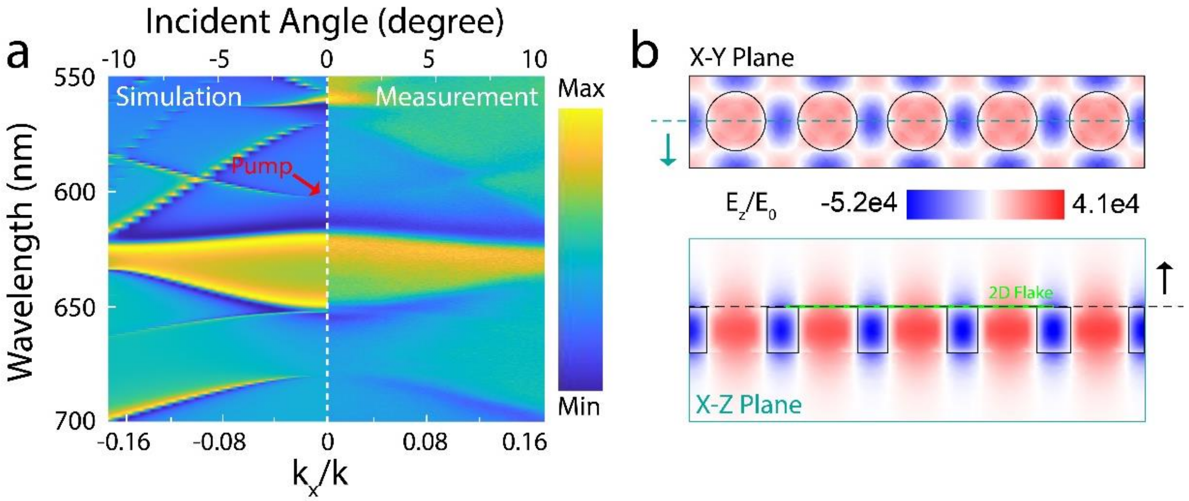

Supplementary Fig. 5: The optical characterization of the PhC slab supports the avoided crossing mode BIC. The PhC slab has the parameters of periodicity, a = 510 nm, radius of holes, r = 102 nm, and thickness of the slab, h = 233 nm. (a) the comparison of the optical band structure between the simulation and the measurement. (b) the $\mathbf{E_z}$ field distribution in the **X-Y** plane and the **X-Z** plane, respectively.

6. The PL emission polarization analysis of the bright and dark excitons.

In order to analyze the PL emission polarization, we draw a schematic to illustrate the polarization mapping of the radiation pattern at the **k**-plane for the out-of-plane and in-plane dipoles, respectively. The out-of-plane dipole should yield a radical polarization distribution at the **k**-plane because of its rotational symmetry along the axis of the objective lens (**Z**-direction) as shown in **Supplementary Fig. 6a**. On the other hand, the corresponding polarization distribution for the in-plane dipole should be along the same direction (**Supplementary Fig. 6b**).

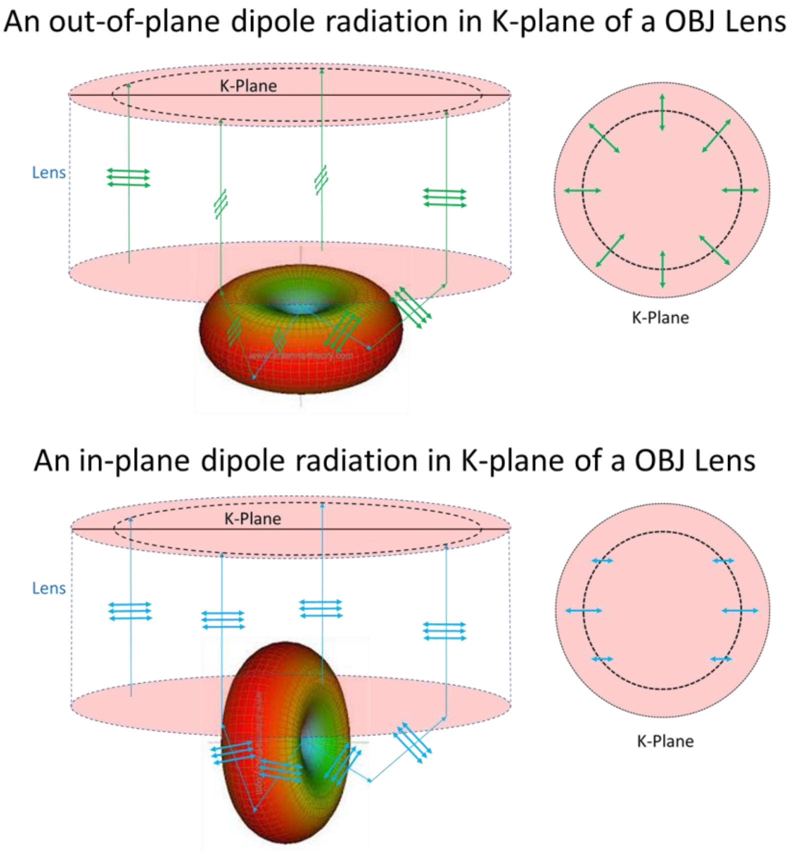

Supplementary Fig. 6: The polarization distribution in the **k**-plane of the PL signal radiated by (a) the out-of-plane oriented dipole (dark excitons) and (b) the in-plane oriented dipole (bright excitons).

7. Formation of the Friedrich-Wintgen BIC

While situated within the energy range of a radiative continuum, BICs possess distinctive characteristics (symmetry, polarization, and so on), forming a momentum-space or phase singularity that makes them intrinsically non-radiative. A plausible way to understand BICs is through destructive interference/coupling between different resonances, modes, or radiative channels. To illustrate the formation of the FW-BIC, we simulate and analyze the normalized out-of-plane electric field ($\mathbf{E}_z$) distribution of the two constituting transverse magnetic (TM) modes (dashed lines) **Supplementary Fig. 7**. The coupling of the TM modes introduces an anti-crossing effect, leading to the two new hybrid bands (upper and lower). Each hybrid band consists of two parts (left and right), and the similar electric field patterns between the upper left/right and lower left/right parts confirm that they are from the same TM modes. More importantly, the electric fields

inside the photonic crystal structure (dashed lines) for the lower left and right parts have opposite polarities (+/− and −/+) and thus are out-of-phase, leading to destructive interference/coupling and generating an FW-BIC. In contrast, the upper left and right parts have in-phase electric fields with the same polarities (+/− and +/−), leading to constructive interference/coupling and causing a leaky mode.

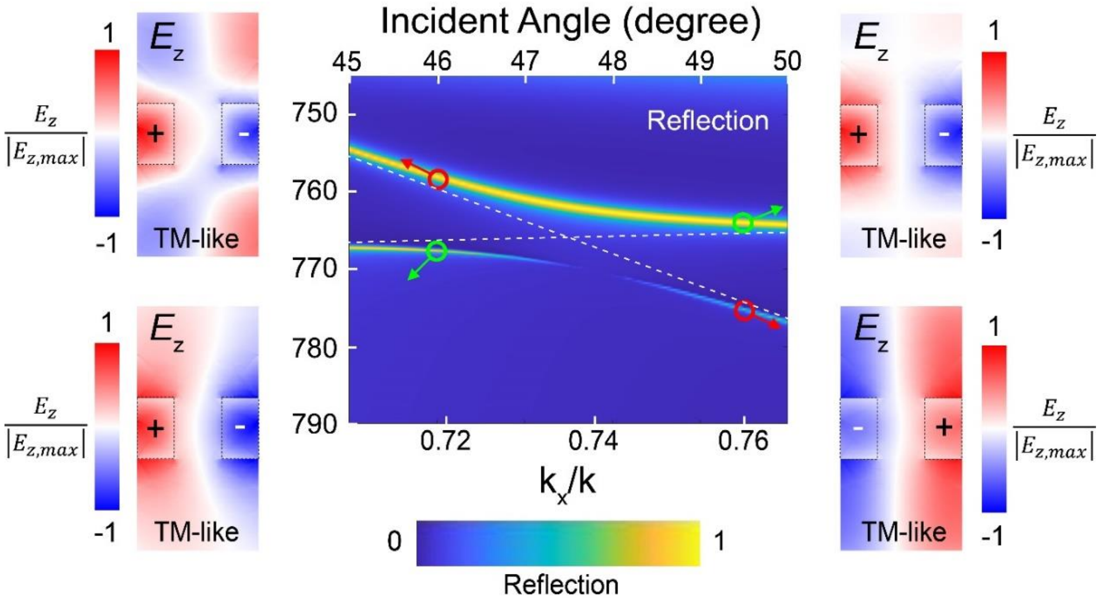

Supplementary Fig. 7: Formation of FW-BICs with mode analysis. The figures at the four corners are the normalized electric field distributions of the upper and lower hybrid photonic bands. The lower left and right distributions show opposite polarities (+/− and −/+) or are out-of-phase, leading to a destructive interference/coupling that generates an FW-BIC. The upper left and right parts, however, have in-phase electric fields with the same polarities (+/− and +/−), leading to constructive interference/coupling that yields a leaky mode.

8. The calculation of the out-of-plane electric field conversion efficiency of the on-Γ BIC

Supplementary Fig. 8 shows how the incident laser beam compressed into the BIC mode in both the K-space and energy space. We used a short-pass edge filter (FESH0700, Thorlabs) and placed it in front of the laser beam with a twist angle. The short-pass filter edge can blue shift to ~694.8 nm. The incident laser can be narrowed in the energy space with an FWHM of 3 nm (**Supplementary Fig. 8c**). In this way, we can compress more energy into the BIC mode (at 694 nm) to gain a higher enhancement factor and $\mathbf{E_z}$ converter efficiency. On the other hand, we used a 0.2X beam expander (GBE05-A, Thorlabs) to squeeze the laser beam in a smaller volume to

compress more energy near 0° to match the on-Γ BIC mode.

Further, we calculated the average enhancement factor of the $|\mathbf{E_z}/\mathbf{E_0}|^2$ at the top surface of the PhC slab (periodicity, a = 450 nm, radius of holes, r = 140 nm, thickness of the slab, h = 265 nm). Note that $\mathbf{E_0}$ is x polarized. We used the ratio between the integral of $|\mathbf{E_z}|^2$ at the top surface of the PhC slab and $|\mathbf{E_0}|^2$ at the same position in the air (removing the PhC slab in simulation). The integral range in wavelengths is 692.5 nm to 695.5 nm and in K-space is -2° to 2°. We assumed the focused laser spot has a gaussian profile and used the rotational symmetry approximation (**Supplementary Fig. 1**). We also calculated the average $|\mathbf{E_x}/\mathbf{E_0}|^2$ and average $|\mathbf{E_y}/\mathbf{E_0}|^2$ and found that the ratio between $|\mathbf{E_z}/\mathbf{E_0}|^2$ and $|\mathbf{E_x}/\mathbf{E_0}|^2$ is ~30.5 where the ratio between $|\mathbf{E_x}/\mathbf{E_0}|^2$ and $|\mathbf{E_y}/\mathbf{E_0}|^2$ is ~5.2. The large $|\mathbf{E_z}/\mathbf{E_0}|^2$ over $|\mathbf{E_x}/\mathbf{E_0}|^2$ ratio means most of the laser energy is in the out-of-plane direction and the large $|\mathbf{E_x}/\mathbf{E_0}|^2$ over $|\mathbf{E_y}/\mathbf{E_0}|^2$ ratio means the photonic crystal maintains the in-plane linear polarization.

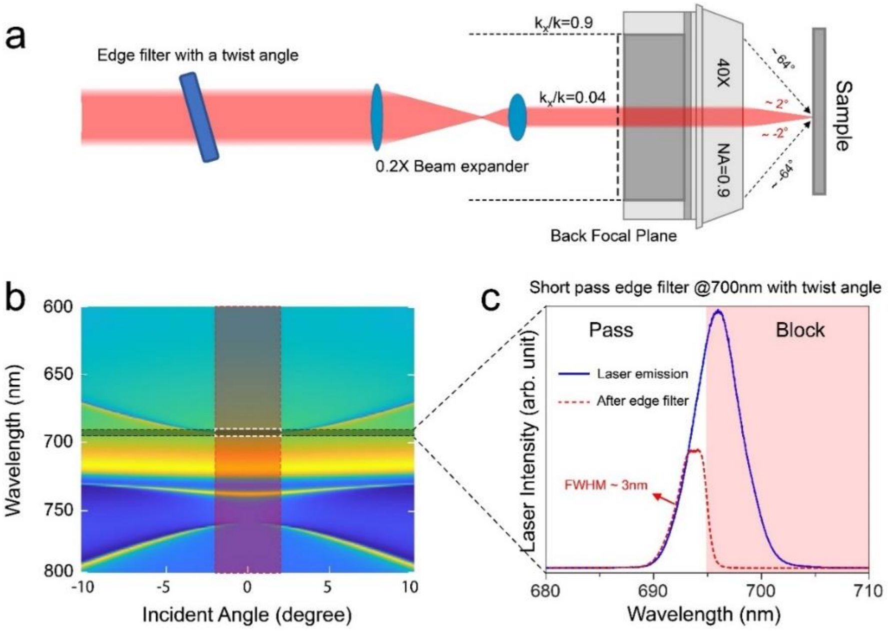

Supplementary Fig. 8: The Incident laser beam focused on the BIC. (a) schematic of the light pass with a short pass edge filter placed with a twist angle (edge at 694.8nm) and 0.2X beam expander which were used to compress the laser beam in **k**-space and energy space. The laser can be compressed into +/- 2° in the **k**-space as shown in the red region in (b), the angle-resolved reflection spectra mapping. (c) The laser can be narrowed in energy space with an FWHM of 3 nm.

9. The calculation of the Purcell factor of the avoid-crossing BIC

The Purcell factor of the dark excitons was calculated using a commercial finite-difference time-domain (FDTD) simulation software (Lumerical). We randomly put a series of out-of-plane or in-plane (x-direction) dipoles at the top surface of the PhC slab. The out-of-plane or in-plane dipoles mimic the dark excitons or bright excitons radiation moments in the WSe_2 monolayer. To minimize the differences between the experiment and the simulation, an h-BN flake with 5 nm of thickness was placed at the top of the PhC slab where dipoles were placed between the PhC slab and the h-BN layer. In FDTD simulations, the Purcell factor is calculated by the ratio of the emission intensity radiated by the out-of-plane or in-plane dipoles on the PhC slab and the emission in the free space (no PhC slabs nor h-BN). We found that most of the dark excitons' emission was radiated through the avoided crossing BIC channel.

In this simulation, A 30-by-30-periods of simulation area and a **Z**-oriented dipole array were used in the simulation. The simulation time was ~20 hours using a computer with the i7-8700 CPU and the 64 GB DDR4 RAM.

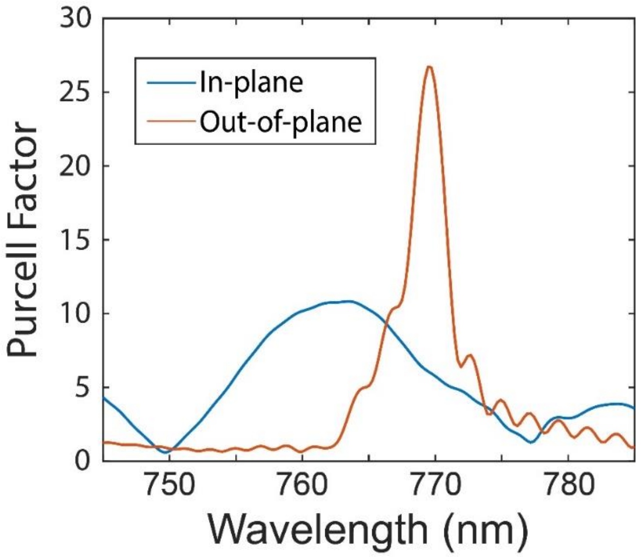

Supplementary Fig. 9: The calculated Purcell factor of the dark excitons (red) and the bright excitons (blue) excited in the WSe_2 monolayer at the top surface of the PhC slab. The PhC slab has the parameters of periodicity, a = 510 nm, radius of holes, r = 102 nm, and thickness of the slab, h = 233 nm.

10. Collection efficiency of the PL emission radiated by bright excitons and dark excitons

In this section, we would like to discuss the collection efficiency of the PL emission radiated by bright excitons and dark excitons. Because of their out-of-plane radiating nature, dark excitons PL emits primarily towards the in-plane direction. Even if a sufficiently large numerical aperture objective lens (as high as 0.9) was used, we still cannot collect the dark exciton emission with high efficiency.

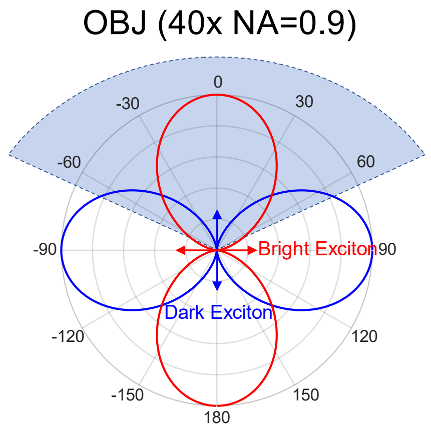

Supplementary Fig. 10: Collection efficiency of the PL emission radiated by bright excitons (red) and dark excitons (blue) using a 40X objective lens with a numerical aperture of 0.9.

11. Bright exciton PL emission decoherence at room temperature

Although the coherence of the bright exciton emission in WSe_2 is well studied at both cryogenic and room temperature[1-3]. We measured the monolayer WSe_2 PL pattern in **k**-space under p-polarized excitation. **Supplementary Figs. 11a, b,** and, **d** show the PL pattern without an analysis polarizer and with co-and cross-direction analysis polarizers. We extracted the intensity profiles from the PL patterns under co- and cross-polarized conditions, respectively (**Supplementary Fig. 11b**). To quantify valley coherence the degree of linear polarization (DOLP) $\rho_l = \frac{I_{co}-I_{cross}}{I_{co}+I_{cross}}$, where l denotes linear polarization, and I is the measured intensity of co- (cross-) polarized PL signal[1]. This resulted in a measured $\rho_l = 7.85\%$ indicating the weak valley coherence of the bright exciton at room temperature[2].

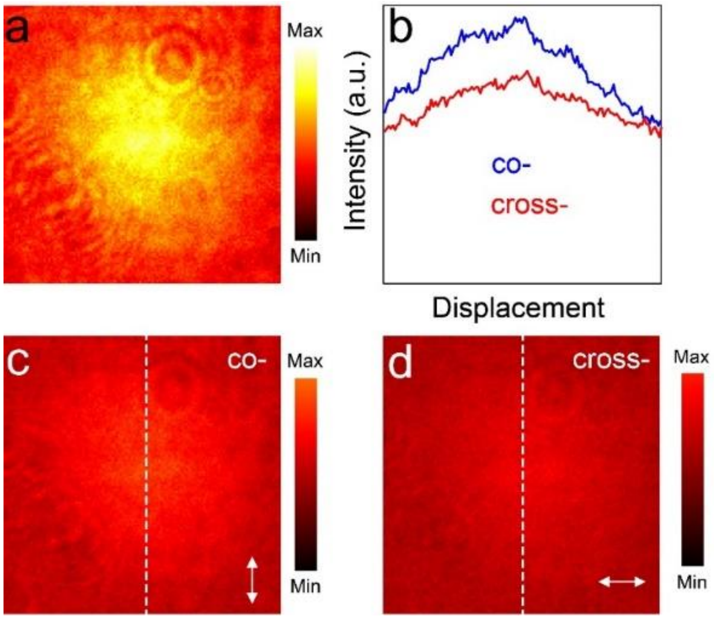

Supplementary Fig. 11: Monolayer WSe_2 bright exciton emission patterns in **k**-space (a) without analysis polarizer, (c) with the co-polarized polarizer, and (d) with the cross-polarized polarizer. (b) The extracted intensity profile from (c) and (d), shows the weak valley coherence of the bright exciton at room temperature. The excitation laser has a wavelength of 650 nm and a pump power of 50 μW.

12. Interference setup using a cylindrical lens

Inspired by the cylindrical lens enabled interference setup for demonstrating vortex beam[4], we used the setup as shown in **Supplementary Fig. 12** to demonstrate our dark excitons directional emission is coherent, i.e., emission spots with opposite phase have destructive interference pattern (**Fig. 5b** in manuscript). The setup is based on our previous spectra analysis mode setup, and we just replaced the L4 Fourier Lens in **Supplementary Fig. 3b** with a cylindrical lens (focal length is 100 mm). In this way, we can make the directional emission spots that toward opposite directions overlap and then can observe their interference pattern.

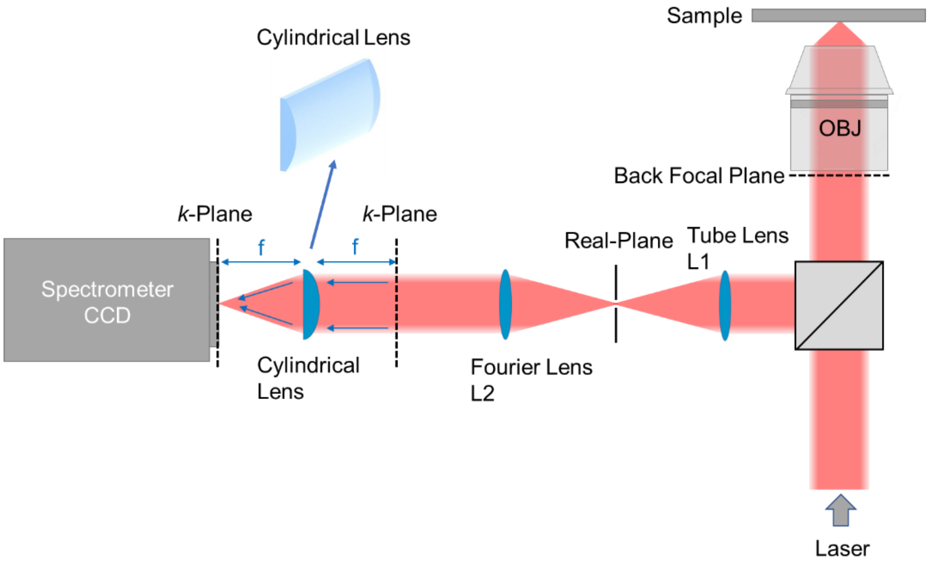

Supplementary Fig. 12: Interference setup enabled by a cylindrical lens. Spatially distributed light waves passing through a cylindrical lens possess spatial interference patterns detectable by a spectrometer. The cylindrical lens (focal length of 100 mm) replaced the Fourier lens L4 with a focal length of 100 mm in **Supplementary Fig. 3b** to enable interference.

13. Optical band structure measurements using halogen lamp and supercontinuum laser

In order to demonstrate the 100-by-100 periods suspend photonic crystal is sufficiently large to guarantee the bound states in the continuum (BICs), we used the supercontinuum laser as the light source to observe the optical band structure. **Supplementary Fig. 13** shows the comparison of measured optical band structure using a halogen lamp and supercontinuum laser. The measured optical band structures in **Fig. 2b**, **Supplementary Fig. 4**, and **Supplementary Fig. 5a** were obtained using a halogen lamp and we have checked the real-space iris to make sure the spectrometer received only the light reflected by the photonic crystal device (**Supplementary Fig. 13a**). As a comparison, we used a supercontinuum laser to carry out a similar measurement. Thanks to the high coherence of the supercontinuum laser, the laser beam can be focused into a very small volume (~10 μm, **Supplementary Fig. 13b**). **Supplementary Fig. 13c** shows the optical band structure comparison using two different light sources (halogen lamp and supercontinuum laser, right and left) and the result shows high agreement.

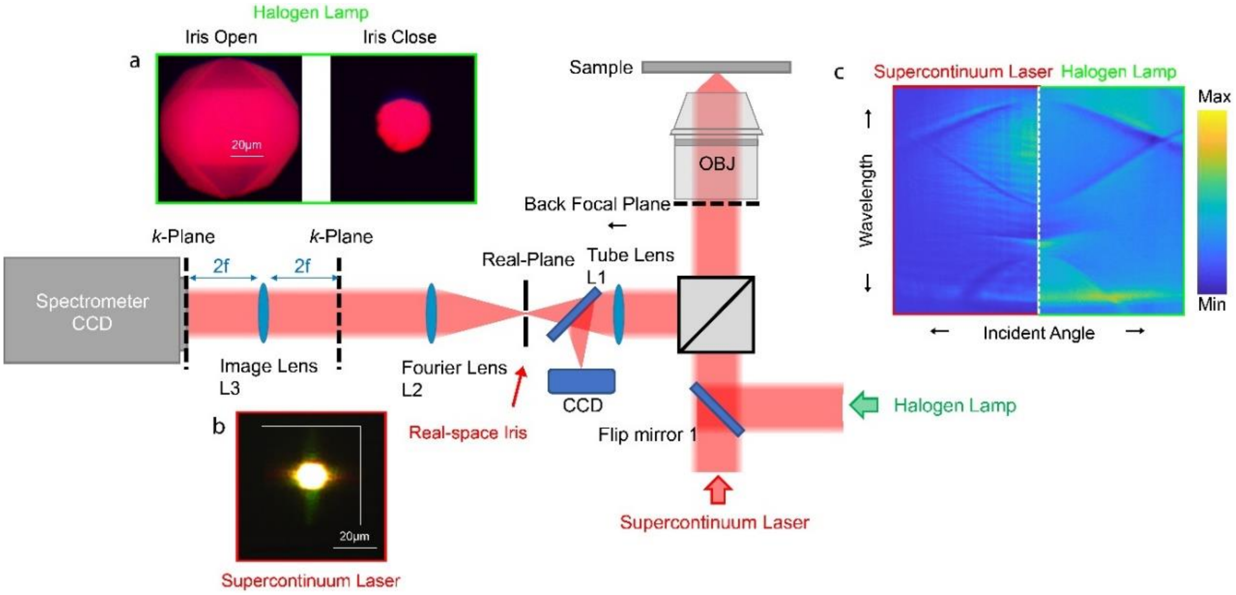

Supplementary Fig. 13: Schematic of the optical band structure measurement setup. The halogen lamp and supercontinuum laser can serve as light sources. (a) the optical microscope image of the device in the real plane using the halogen lamp as a light source, the closed iris allows the light reflected from the device part to pass only. (b) the optical microscope image of the device using the supercontinuum laser as a light source, a small region of the device reflects the incident light. (c) the optical band structure comparison between two light sources.

14. Microscopy image of the tightly focused gaussian spot

Supplementary Fig. 14 show an optical microscope image of the tightly focused beam using the 40X objective lens with a numerical aperture (NA) of 0.9 for the dark exciton pump in this work. We measured the focused beam size as shown in **Supplementary Fig. 14** and the Full Width at Half Maximum (FWHM) of this Gaussian spot is 6.86 μm. It is worth noting that we used the 0.2X beam expander to compress the laser beam in **k**-space into +/- 2°(more details see **Supplementary Fig. 8**). The smaller incident beam would result in a larger focused spot.

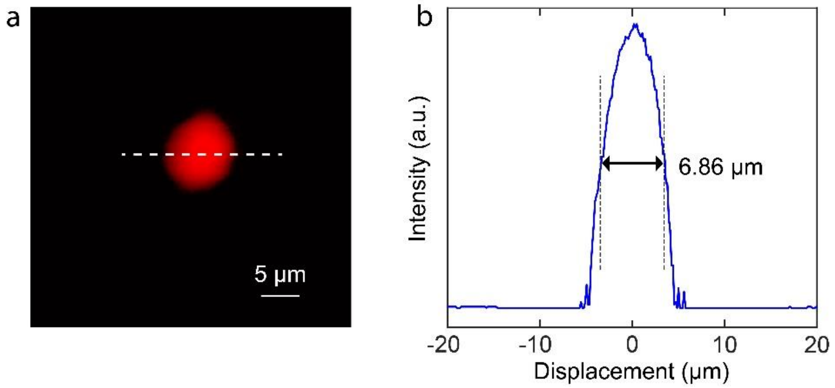

Supplementary Fig. 14: Microscopy image of the tightly focused gaussian spot. The Full Width at Half Maximum (FWHM) of this Gaussian spot is 6.86 μm.

15. The measured refractive index of LPCVP Si_3N_4 film.

We used the commercially available silicon nitride /silicon wafer (Silicon Valley Microelectronics, SVM). The 300nm thick silicon nitride layer was deposited on the silicon wafer with low-pressure chemical vapor deposition (LPCVD). They claimed the silicon nitride film thickness was 3,000 Å (300 nm) and the refractive index is 2.30 +/- 0.05 at 632.8 nm. In addition, the refractive index of silicon nitride with excellent stress control should be around 2.2-2.3[5]. **Supplementary Fig. 15** shows the Si_3N_4 refractive index as a function of the wavelength.

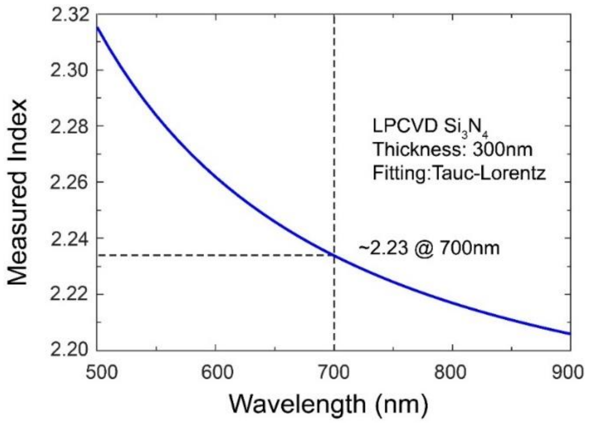

Supplementary Fig. 15: The measured refractive index of LPCVP Si_3N_4 film.

16. Influence of the WSe_2 monolayer

Although the WSe_2 (or other WSe_2-like 2D materials) monolayers are semiconductors, their image part of refractive index (κ) is not negligible which may bring large damping for photonic

devices. We carried out the comparison simulations for the photonic crystal with and without the WSe_2 monolayer. Because of the big dimension mismatch between the thickness of WSe_2 (~0.7 nm) and the periodic of photonic crystal (450 nm), the numerical simulation (COMSOL and FDTD) requires large RAM to render the mesh for accurate calculations. We estimated two different oblique angles (1° and 3°) in this simulation. Even with a high-end computer (it has two Intel Xeon Gold 6150 CPUs and 384 GB DDR4 RAM), simulation of one spectrum still requires several days.

Supplementary Fig. 16b shows the reflection spectra comparisons for photonic crystals with and without the WSe_2 monolayer. The introduction of the thin layer would make the Fano resonance redshift for about 1.5 nm. The refractive index of WSe_2 was extracted from Ref. [6]. This redshift can be compensated by our strategy discussed in Supplementary section 17 (**Supplementary Fig. 17**). The biggest problem is the large damping of the monolayer will break the **Z**-symmetry of the photonic crystal and result in a largely reduced Q-factor (**Supplementary Figs. 16c** and **d**). In this work, the on-Γ BIC converts the normal incident laser with in-plane polarization into near-field energy with out-of-plane polarization and has a giant enhancement. We further checked the conversion efficiency of the degenerated on-Γ BIC to make sure the lower Q-factor resonances still can serve as the efficient convertor. **Supplementary Fig. 16e** shows the absolute value of out-of-plane electric field ($\mathbf{E_z}$) distributions when the incident angle is 1°. It is worth noting that the two Fano resonances shown in **Supplementary Fig. 16e** have different wavelength scales and we just want to label the positions for each **E**-field distribution. Although the Fano resonance in the case without WSe_2 has higher **E**-field intensity (the highest intensity at the 3rd position, we used its maximum intensity to do the normalization for each **E**-field distribution), its smaller peak width has a lower chance to enhance the incident laser (5 nm width in wavelength). We use the equation shown below to compare the conversion efficiencies of two cases.

$$R = \frac{\int_{\lambda_0-1/2\Delta\lambda}^{\lambda_0+1/2\Delta\lambda} ave(\varepsilon_1 E_z(1))d\lambda}{\int_{\lambda_0-1/2\Delta\lambda}^{\lambda_0+1/2\Delta\lambda} ave(\varepsilon_2 E_z(2))d\lambda}$$

where the λ_0 demotes the center wavelength of the pump, $\Delta\lambda$ is the wavelength width of the pump, $\mathrm{ave}(\mathbf{E_z})$ denotes the average of the out-of-plane electric field intensity in the **X-Y** plane at the top surface of the photonic crystal, and ε_1 or ε_2 are the relative dielectric constant for each case (with or without WSe_2). The ratio R (1°, with over without WSe_2) is 2.53, and the ratio R (3°) is 0.76 when the wavelength width of the pump is 5 nm, indicating the lower Q-factor has higher overall

covert efficiency, counterintuitively.

In summary, the WSe_2 monolayer has a relatively large image part of the refractive index, which will largely reduce the Q-factor of the on-Γ BIC. It is true that a higher Q-factor of the Fano resonance has a larger electric field intensity, the sharp resonance peak, however, will reduce the coupling efficiency between resonance and pump laser, which would decrease the overall efficiency.

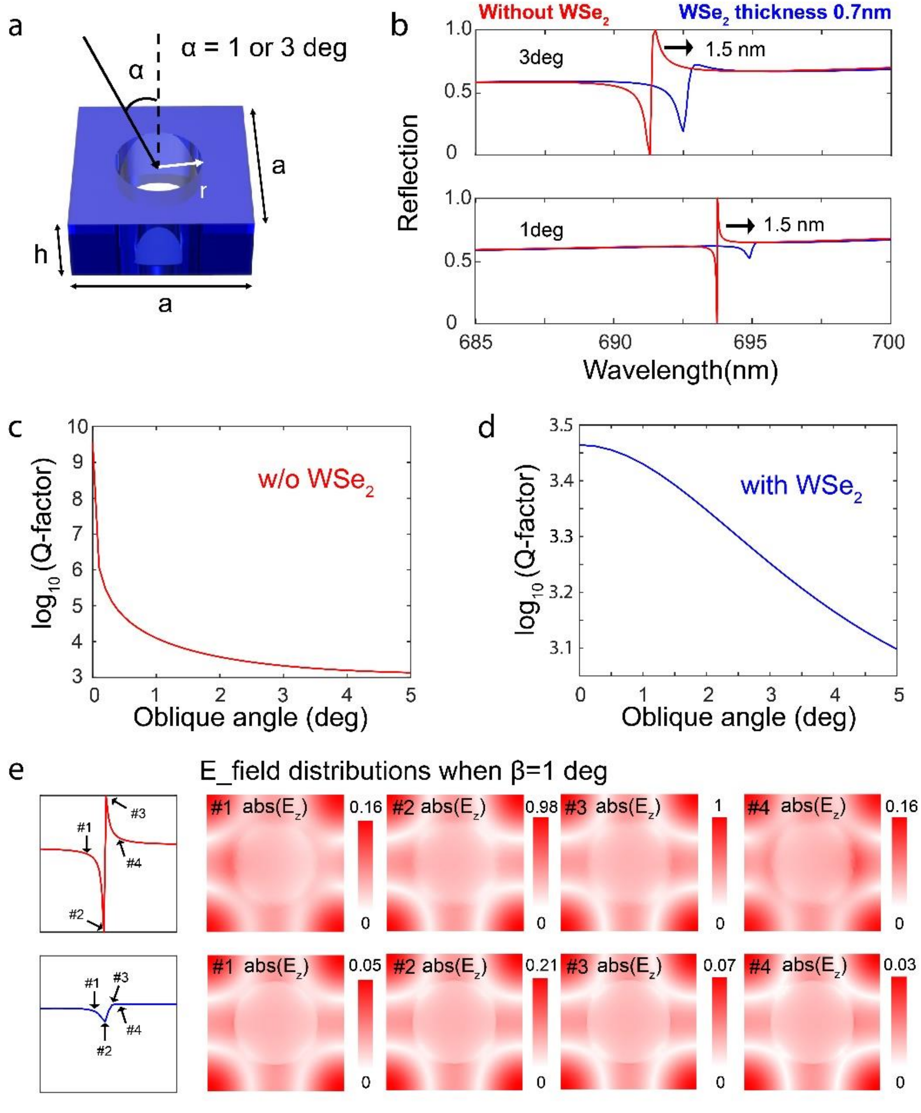

Supplementary Fig. 16: The integration of the WSe_2 monolayer influences the photonic crystal. (a) schematic of the simulation. (b) reflection spectra of a photonic crystal with and without WSe_2 monolayer cases, respectively. A 1.5 nm red shift will be introduced for the integration of WSe_2. (c, d) Q-factors of photonic crystal devices for with and without WSe_2 monolayer cases, respectively. (e) Electric field distributions when the incident angle is 1°.

17. Influence of h-BN flake and compensation strategy

Supplementary Fig. 17 shows the comparison between the designed structure and the h-BN flake on top with compensation. Thanks to the refractive index of h-BN being close to the silicon nitride, this strategy works very well and only a 0.15 nm difference in the resonance wavelength was observed. We found that the **Z**-symmetry was slightly broken by the h-BN layer but the Fano resonances with high-Q-factor were still supported (**Supplementary Fig. 17c**). To note that the simulation for h-BN with 5 nm thickness requires large RAM (~75 GB RAM with h-BN and ~5 GB RAM for original structure) and quite a longer simulation time (25 mins for one wavelength point with h-BN and ~3 s for original structure) using COMSOL 5.5. Our COMSOL computer has two Intel Xeon Gold 6150 CPUs and 384 GB DDR4 RAM. Because of the extremely long simulation time (~25mins for each point, more than 200 points for each spectrum) even using the powerful simulation computer, we just examined one reflection spectrum with an oblique angle of 3°. We used the eigenfrequency model in COMSOL to estimate the Q-factor with different oblique angles with and without an h-BN flake. Interestingly, the structure with the h-BN layer gives a slightly higher Q-factor for different oblique angles.

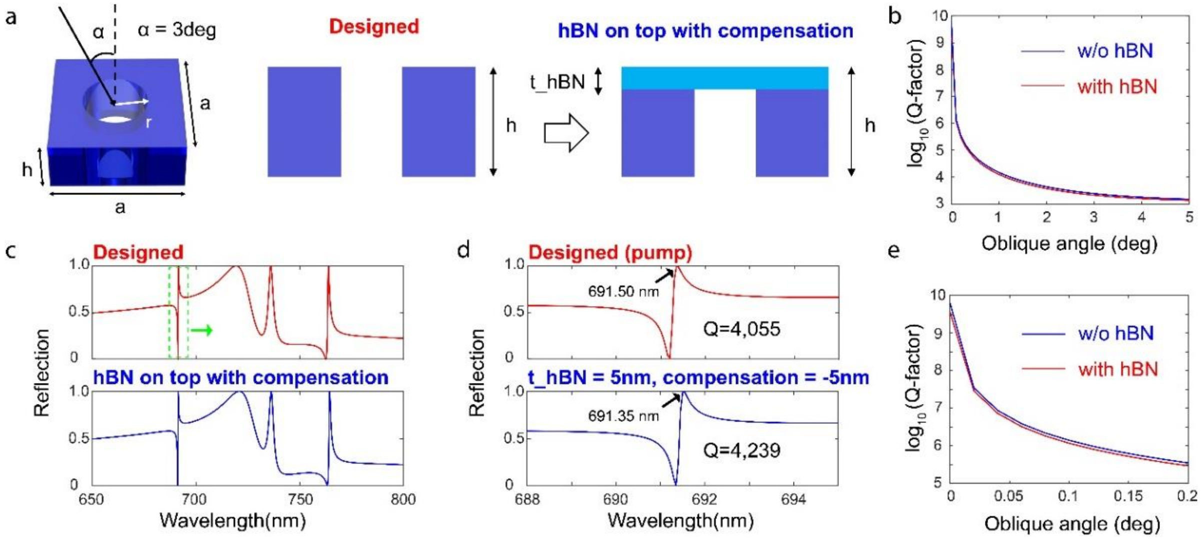

Supplementary Fig. 17: The integration of h-BN flake influence on the photonic crystal and the compensation strategy. (a) schematic of the simulation. (b, e) Q-factors of photonic crystal devices for with and without h-BN flake cases, respectively. (c, d) reflection spectra comparisons of photonic crystal devices with and without h-BN flake cases, respectively.

18. Modal overlap between on-Γ BIC and FW-BIC

We estimated the modal overlap (also known as the mode-matching factor) between on-Γ BIC and FW-BIC used in this work. We used the eigenfrequency model in COMSOL to extract the modes of the two BICs and used the equation below for the modal overlap estimation.

$$\eta = \frac{|\int E_1^* * E_2 dA|^2}{\int |E_1|^2 dA * \int |E_2|^2 dA}$$

We would like to note that $\mathbf{E_1}$ and $\mathbf{E_2}$ are absolute values instead of the original value[7]. In the normal case for modal overlap, the energy transfer would happen to two different modes directly. However, in this case, the WSe_2 monolayer serves as the medium to connect the two modes. Specifically, excitons can be excited because of the energy in the first mode and then recombined resulting in a photoluminescence (PL) signal. The PL signal is coupled with the second mode and can be directionally emitted. In the whole process, only the absolute value of the **E**-field contributes to the mode coupling whereas the phase of the **E**-field does not. Thus, we used the absolute value of the **E**-field for the modal overlap calculation. The distribution of the absolute value of the E-field for two modes is shown in **Supplementary Fig. 18**. The modal overlap is 45.73%.

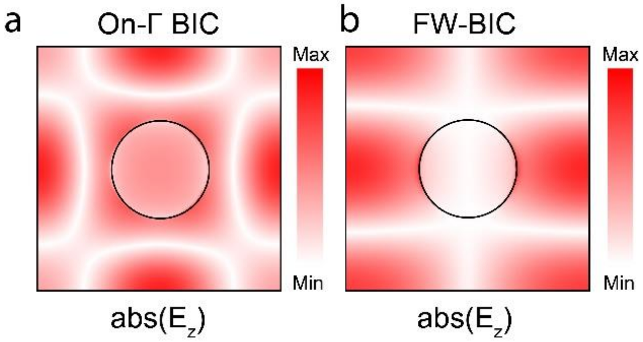

Supplementary Fig. 18: Electric field distributions in the **X-Y** plane at the top surface of the photonic crystal device of on-Γ BIC and FW-BIC.

19. Comparison of different dark excitons brightening methods.

Compared with current state-of-the-art dark excitons brightening methods, the method in this work has both high brightening efficiency and high collection efficiency, thus the high overall efficiency. The method in this work also demonstrated room temperature and CMOS compatibility.

Method	Schematic of method	Brightening efficiency (a)	Collection efficiency (b)	Overall efficiency (a*b)	Enhancement	Temperature condition	Directional emission	CMOS compatible
In-plane Magnetic field ($B_{\parallel}$)	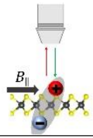 $B_{\parallel}$[8]	Weak	Weak	Weak	No	Low T	No	No
Out-of-plane Electric field ($E_{\perp}$)	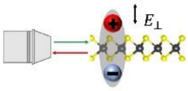 Edge detection[9]	Weak	**High**	Weak	No	Low T	No	No
	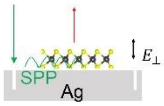 SPP[10]	Medium	Weak	Weak	No	Low T	No	No
	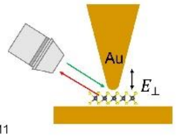 TEPL[11]	**High**	Medium	Medium	**Yes**	**Room T**	No	No
	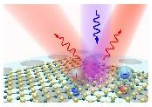 BIC (This work)	**High**	**High**	**High**	**Yes**	**Room T**	**Yes**	**Yes**

Supplementary Fig. 19: Comparison of different dark excitons brightening methods. Other works include in-plane magnetic field[8], edge detection[9], surface plasmon polariton (SPP) coupling[10], and Tip-Enhanced Photoluminescence (TEPL)[11].

Reference

1. Qiu, L., Chakraborty, C., Dhara, S. & Vamivakas, A. Room-temperature valley coherence in a polaritonic system. *Nat. Commun.* **10**, 1513 (2019).

2. Jones, A. M. et al. Optical generation of excitonic valley coherence in monolayer WSe_2. *Nat. Nanotechnol.* **8**, 634-638 (2013).

3. Hao, K. et al. Direct measurement of exciton valley coherence in monolayer WSe_2. *Nat. Phys.* **12**, 677-682 (2016).

4. Shvedov, V. G. et al. Efficient beam converter for the generation of high-power femtosecond vortices. *Opt. Lett.* **35**, 2660-2662 (2010).

5. Yang, C. & Pham, J. Characteristic study of silicon nitride films deposited by LPCVD and PECVD. *Silicon* **10**, 2561-2567 (2018).

6. Hsu, C. et al. Thickness-dependent refractive index of 1L, 2L, and 3L MoS_2, $MoSe_2$, WS_2, and WSe_2. *Adv. Opt. Mater.* **7**, 1900239 (2019).

7. Yu, R., Wang, C., Benabid, F., Chiang, K. S. & Xiao, L. Robust mode matching between structurally dissimilar optical fiber waveguides. *ACS Photonics* **8**, 857-863 (2021).

8. Zhang, X.-X. et al. Magnetic brightening and control of dark excitons in monolayer WSe_2. *Nat. Nanotechnol.* **12**, 883-888 (2017).

9. Wang, G. et al. In-plane propagation of light in transition metal dichalcogenide monolayers: optical selection rules. *Phys. Rev. Lett.* **119**, 047401 (2017).

10. Zhou, Y. et al. Probing dark excitons in atomically thin semiconductors via near-field coupling to surface plasmon polaritons. *Nat. Nanotechnol.* **12**, 856-860 (2017).

11. Park, K.-D., Jiang, T., Clark, G., Xu, X. & Raschke, M. B. Radiative control of dark excitons at room temperature by nano-optical antenna-tip Purcell effect. *Nat. Nanotechnol.* **13**, 59-64 (2018).
